# Associations between Chronic Kidney Disease and Migraine Incidence: Findings from a Korean Longitudinal Big Data Study

**DOI:** 10.3390/jpm14040356

**Published:** 2024-03-28

**Authors:** Mi Jung Kwon, Jwa-Kyung Kim, Min-Jeong Kim, Dae Myoung Yoo, Na-Eun Lee, Kyeong Min Han, Nan Young Kim, Ho Suk Kang, Hyo Geun Choi, Eun Soo Kim

**Affiliations:** 1 Department of Pathology, Hallym University Sacred Heart Hospital, Hallym University College of Medicine, Anyang 14068, Republic of Korea; mulank99@hallym.or.kr; 2 Laboratory of Brain and Cognitive Sciences for Convergence Medicine, Hallym University College of Medicine, Anyang 14068, Republic of Korea; ydm@hallym.ac.kr (D.M.Y.); D23009@hallym.ac.kr (N.-E.L.); km.han@hallym.ac.kr (K.M.H.); 3 Division of Nephrology, Department of Internal Medicine, Hallym University Sacred Heart Hospital, Hallym University College of Medicine, Anyang 14068, Republic of Korea; kjk816@hallym.or.kr; 4 Department of Radiology, Hallym University Sacred Heart Hospital, Hallym University College of Medicine, Anyang 14068, Republic of Korea; drkmj@hallym.or.kr; 5 Hallym Data Science Laboratory, Hallym University College of Medicine, Anyang 14068, Republic of Korea; 6 Hallym Institute of Translational Genomics and Bioinformatics, Hallym University Medical Center, Anyang 14068, Republic of Korea; honeyny@hallym.or.kr; 7 Division of Gastroenterology, Department of Internal Medicine, Hallym University Sacred Heart Hospital, Hallym University College of Medicine, Anyang 14068, Republic of Korea; hskang76@hallym.or.kr; 8 Suseo Seoul E.N.T. Clinic, 10, Bamgogae-ro 1-gil, Gangnam-gu, Seoul 06349, Republic of Korea; mdanalytics@naver.com

**Keywords:** chronic kidney disease, migraine risk, longitudinal follow-up study, national health big data

## Abstract

While headaches frequently occur in individuals with chronic kidney disease (CKD), there are few statistical evaluations of their connection to migraines in population-based studies. In this nationwide longitudinal follow-up study of Korean health examination data (2002–2019), a total of 15,443 participants with CKD and 61,772 matched controls were enrolled. We applied overlap-weighted Cox proportional hazard regression models to assess hazard ratios, examining the correlation between CKD and the development of migraines. After accounting for various factors, we observed a modest reduction of approximately 11% in the likelihood of migraine occurrence among CKD patients (95% confidence intervals = 0.81–0.97) during the 16-year monitoring period. Subgroup analysis revealed a significant association among specific demographic and health conditions, including individuals aged 70 or older, females, overweight individuals, nonsmokers, and those without hypertension or diabetes. Our research may indicate a potential relationship between CKD and the onset of migraines in Korean adults, suggesting a slight reduction in the probability of the occurrence of migraines among those with CKD. These findings emphasize the need for attentive follow-up and preventive management in individuals without the identified protective factors, particularly in male CKD patients under the age of 70 with hypertension.

## 1. Introduction

Chronic kidney disease (CKD), characterized by progressively declining kidney function and vascular complications, can ultimately lead to chronic renal failure that requires dialysis or transplantation [1]. CKD often gives rise to various neurological issues, including cerebrovascular disorders such as stroke, cognitive impairment, white matter diseases, and headaches [2,3,4,5]. These neurological complications are part of the heightened risk of cardiovascular events correlated with CKD, contributing to a significant 41.5% increase in global CKD mortality rates across all age groups [6,7]. The overall national incidence of CKD is 6.2% in Korea [8], which is lower than that in other countries (10–13%) [9]. On the other hand, migraines, affecting approximately 10% of the population, particularly women and urban dwellers aged 25–64, are severe unilateral throbbing headaches with possible accompanying symptoms such as nausea, light, and sound sensitivity [10]. Although more prevalent in younger individuals, those older than 55 years still have a considerable migraine incidence rate of approximately 20–34% [11]. Migraines are divided into two types: with and without aura, the former featuring preheadache visual or sensory changes [10]. The overall prevalence of migraine in Korea is 6.1% [12]. In Asian countries, the reported prevalence of migraine varies from 1% to 22%, which is relatively lower than the rates observed in North American countries and European countries (10–25%) [13]. Although both the prevalence of CKD and migraines in Korea seem to be lower than those in other countries, CKD and migraines are both prevalent and impactful health conditions, presenting significant challenges in both public health and clinical practice worldwide [7,9] and in Korea [8,14].

Cumulative research suggests a bidirectional relationship between CKD and migraines, aligning with theories of kidney–nervous system communication [15,16]. Considering that both the kidneys and the brain are considered end organs, they share comparable anatomical vascular systems and hemodynamic characteristics, rendering them susceptible to vascular harm [15,16]. Hence, it is plausible that CKD could contribute to the development of migraines. Nevertheless, the longitudinal nature of their association remains inadequately understood, especially in terms of epidemiologic evidence. The specific link between CKD and migraines has been relatively underexplored in cohort studies, leading to conflicting findings [17,18]: a study from Taiwan reported a 22% enhanced risk of subsequent occurrence of CKD in the migraine group [17], while a study conducted in the UK population found no causal effect of migraines on the development of CKD [18]. Notably, these two cohort studies [17,18], along with the majority of previously published reviews exploring the relationship between these two conditions, have predominantly focused on the question of migraines as a risk factor for the initiation of CKD [5,17,19]. While headaches are commonly experienced by individuals with CKD [2], there is a notable lack of statistical evaluations within population-based studies that specifically investigate the relationship between CKD and incident migraines, raising questions about whether CKD may be one of the risk comorbidities inducing migraines in the Korean population. Given the health and economic burdens posed by both CKD and migraines in Korea’s aging and urbanizing population [20], investigating the potential impact of CKD as a risk factor for incident migraines is imperative. Evidence-based management is essential for mitigating potential risks associated with comorbid conditions, including migraines, in individuals with CKD.

Therefore, we hypothesized that the incidence of migraines in the Korean population may be influenced by the presence of CKD. This study expanded upon prior research in the field by concentrating on the specific issue of the impact of CKD on the development of migraines. To enhance the precision of our analysis, we utilized a nationwide health screening cohort, with a primary focus on mitigating the influence of confounding variables, including sociodemographic, lifestyle, and comorbidity factors. We successfully achieved a balance in baseline characteristics between the patient and control groups. Furthermore, we conducted an extensive 16-year nationwide database surveillance analysis to comprehensively explore the potential association between CKD and migraine development.

## 2. Patients and Methods

### 2.1. Study Design

The study acquired approval from the institutional ethics committee (IRB No. 2019-10-023) without requiring written informed consent per the Institutional Review Board guidelines due to secondary information.

The authors harnessed information from the Korean National Health Insurance Service-Health Screening Cohort (KNHIS-HSC), a resource that supplies deidentified, population-based electronic files for research purposes, safeguarding the anonymity of the Korean population, as previously outlined [21]. The diagnostic and treatment codes used in this study conformed to the International Classification of Diseases, 10th Revision, Clinical Modification (ICD-10-CM).

Utilizing the KNHIS-HSC dataset, we included individuals aged 40 years and above spanning medical claim codes from 2002 to 2019, resulting in a dataset of 514,866 adult patients with 895,300,177 medical claim codes. Among these, 17,478 individuals were identified as CKD patients. A control group of 497,388 individuals without CKD diagnosis between 2002 and 2019 was formed, excluding those diagnosed with CKD in 2002 (*n* = 536) to allow for a 1-year washout period. Further refinement involved excluding CKD participants lacking recorded values for blood pressure (*n* = 1), fasting blood glucose (*n* = 2), or body mass index (BMI) (*n* = 2), as well as control group participants assigned the ICD-10 code N18 (*n* = 560).

In the data cleaning process, patients with missing data, CKD diagnosed only once, or migraine diagnosed before CKD were excluded. Stratifying the population into 200 strata based on age (10 categories with a 5-year interval), sex, income (5 categories), and region (urban/rural), we conducted a 1:4 matching process using propensity scores, removing unmatched patients. Covariates were handled by using the values closest to the index date and the data were analyzed with weighted adjustments based on propensity scores. The Charlson Comorbidity Index (CCI) was used to evaluate disease burden and multivariable logistic regression and Cox proportional hazard regression with overlap weighting were applied to minimize confounding factors.

**Figure 1:**
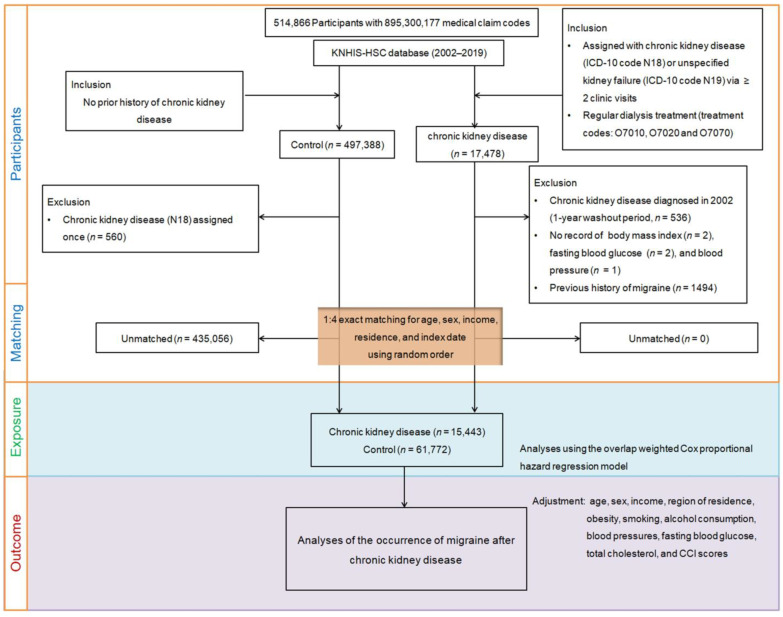
Illustration of the participant selection process utilized in this study. From the initial pool of 514,866 participants within the Korean National Health Insurance Service-Health Screening Cohort (KNHIS-HSC) database, a meticulous matching process resulted in 15,443 patients with chronic kidney disease being matched with 61,772 control participants based on age, sex, income, and region of residence.

Control subjects were randomly chosen to match CKD subjects, aligning index dates for simultaneous assessments. Individuals with a history of migraines prior to the index date were removed from both groups. A total of 15,443 participants with CKD were expertly matched with 61,772 individuals in the comparison group at a 1:4 ratio, with rigorous selection criteria applied. Newly diagnosed migraine cases were identified within both groups from each individual’s index date up to the end of 2019 (Figure 1).

### 2.2. Exposure (CKD) and Outcome (Migraine)

CKD was defined as either receiving a diagnosis of CKD (ICD-10 code N18) or being diagnosed with unspecified renal failure on at least two separate occasions, with the inclusion of individuals undergoing routine dialysis treatment, including hemodialysis and/or peritoneal dialysis, if their records contained relevant therapeutic codes (O7010, O7020, and O7070) [4].

Our definition of migraine relies on the presence of the ICD-10 code (G43) and whether individuals need a cure for their symptoms. Migraine patients with a consistent occurrence of the ICD-10 code (G43) assigned two or more times by neurology specialists were included in the study [22,23]. We excluded participants who had been diagnosed with other types of headaches at the baseline, identified by the ICD-10 code G44. Within this group, individuals diagnosed or treated with the code G431 were classified as having migraine with an aura, while the rest of the subjects with migraines were categorized as having migraines without an aura.

### 2.3. Covariates

The study divided people into 10 age brackets, each spanning a 5-year interval, and categorized them into five income brackets. The residential areas of the participants were categorized as either urban or rural, employing the same methodology as in previous studies. Furthermore, this study utilized a categorization approach consistent with that used in previous research for three variables: smoking, alcohol consumption, and obesity [24]. Obesity was determined on the basis of participants’ BMI in kg/m^2^. Additionally, the study incorporated information on various health indicators, including systolic and diastolic blood pressure levels (measured in mmHg), total cholesterol levels (measured in mg/dL), and fasting blood glucose levels (measured in mg/dL).

In this study, the CCI, a widely utilized tool for evaluating individuals’ overall disease burden by considering 17 different comorbid conditions, was used to assign scores on the basis of the severity and number of diseases, excluding CKD (ICD-10 codes, N18 and N19) from the CCI calculation. The CCI was employed as a continuous variable, encompassing a score ranging from 0, indicating the absence of comorbidities, to 29, representing the existence of diverse comorbidities [25,26]. The selection of covariates for adjustment was based on a review of the literature indicating potential confounders in the association between CKD and migraine incidence [2,3,4,5,17,18,19], ensuring a robust statistical analysis.

### 2.4. Statistical Analyses

Propensity score overlap weighting was employed to balance covariates and increase the effective sample size. The propensity score was calculated using multivariable logistic regression with all covariates. CKD participants were weighted based on the propensity score’s probability, while control participants were weighted based on the probability of one minus the propensity score. This weighting, ranging from zero to one, aimed to achieve optimal balance and enhance analysis precision [27,28].

We used standardized differences to compare general attributes between the CKD and control groups before and after weighting. Effectiveness was assessed by comparing the absolute standardized differences of covariates. An absolute difference below 0.20 indicated a satisfactory balance [29].

We calculated crude incidence rates by dividing the number of events by total person-years and expressed them as occurrences per 1000 person-years. We used Kaplan–Meier and log-rank tests to compare cumulative migraine incidence between the CKD and control groups.

To account for confounding factors and estimate hazard ratios (HRs) with 95% confidence intervals (CIs) for migraine incidence among CKD patients, we used Cox proportional hazard regression with overlap weighting. We analyzed both unadjusted and overlap-weighted models, adjusting for all the mentioned covariates.

We conducted the statistical analyses using SAS software (version 9.4, SAS Institute Inc., Cary, NC, USA). All analyses were two-tailed and significance was defined as *p* < 0.05.

## 3. Results

We matched 15,443 CKD patients with 61,772 controls and compared their baseline characteristics before and after weighted propensity score matching to ensure comparability (Table 1).

Before adjustment, no significant differences were observed between the CKD group and the comparison group for most variables, with standardized differences less than 0.20. However, the CKD group had higher fasting blood glucose and CCI scores than the control group, with standardized differences of 0.30 and 0.54, respectively.

After implementing overlap-weighted adjustments, demographic characteristics, including fasting blood glucose levels and CCI scores, became more comparable between the two groups (all <0.2 for variables), signifying an improved balance in demographic and health-related attributes.

### 3.1. Newly Occurring Migraine Patients in the CKD and Control Groups

Migraine was identified in 349 individuals (2.26%) in the CKD group (*n* = 15,443) and 1901 individuals (3.08%) in the control group (*n* = 61,772). During the 16-year period (2003–2019), the study included 65,152 person-years in the CKD group and 317,925 in the comparison group, showing lower migraine incidence rates in the CKD group (5.36 per 1000 person-years) than in the non-CKD group (5.98 per 1000 person-years), both in crude and overlap-weighted models.

**Figure 2:**
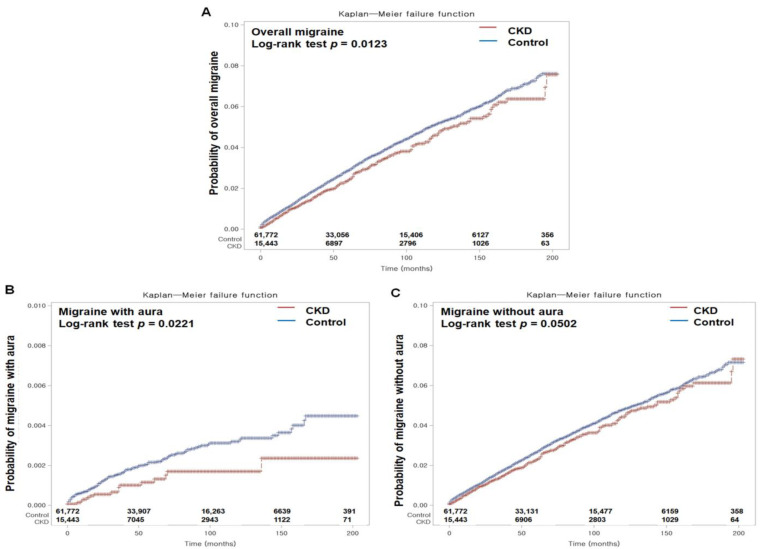
Kaplan–Meier incidence probabilities for overall migraines (**A**) and the subtypes, including migraines with aura (**B**) and migraines without aura (**C**), were assessed in patients with chronic kidney disease (CKD) and control populations older than 16 years from the index date.

Cox proportional hazard analysis revealed that CKD individuals had a reduced likelihood of developing migraines compared with individuals in the control group according to both unadjusted (HR 0.86; 95% CI 0.77–0.97; *p* = 0.012) and adjusted (aHR 0.89; 95% CI 0.81–0.97; *p* = 0.006) models during the 16-year follow-up, even after accounting for demographic and lifestyle factors and medical comorbidities (Table 2). K–M analysis with the log-rank test also revealed a lower incidence of migraine in CKD patients throughout the follow-up period (*p* = 0.0123; Figure 2A).

In our subgroup analyses, we observed that CKD was consistently correlated with a reduced likelihood of migraines across various demographic and health conditions. This significant association was evident in individuals older than 70 years, females, those in the low-income bracket, rural residents, overweight individuals, nonsmokers, and those who drank alcohol less than once a week. Additionally, participants with systolic blood pressure under 140 mmHg, diastolic blood pressure under 90 mmHg, total cholesterol levels of 200 mg/dL or higher, fasting blood glucose levels below 100 mg/dL, and a CCI score of 2 or more also had a lower probability of experiencing migraines (Supplementary Materials: Table S1).

### 3.2. Relationship between CKD and Migraine in Patients with or without Aura

In our study, we investigated the relevance of CKD to the incidence of migraines, both with and without aura. During the follow-up, the CKD group had lower incidence rates of migraine with aura than the control group and this trend persisted in both the crude and adjusted models (0.55 [95% CI 0.33–0.92, *p* = 0.024] and 0.63 [95% CI 0.44–0.92, *p* = 0.016], respectively) (Table 3). K–M analysis with the log-rank test also revealed a lower likelihood of newly occurring migraines with aura in the CKD group than in the control group throughout the monitoring period (*p* = 0.0221; Figure 2B). Subgroup analyses indicated a notably reduced likelihood of developing migraines with aura in specific CKD subgroups, including older adults, females, high-income earners, and others with certain health characteristics (Supplementary Materials: Table S2).

Conversely, for patients with migraines without aura, the CKD group also exhibited lower incidence rates (Table 4). This finding was particularly evident in the adjusted model (aHR 0.90; 95% CI 0.83–0.99; *p* = 0.029). K–M analysis suggested a trend toward a lower cumulative probability of migraines without aura in the CKD group throughout the monitoring period (*p* = 0.0502; Figure 2C). Similar to migraines with aura, subgroup investigations for migraines without aura in the CKD group showed a reduced likelihood across various demographic and health-related categories (Supplementary Materials: Table S3).

## 4. Discussion

In our current investigation, we observed that both overall migraine cases and specific subtypes (including those with and without aura) exhibited slightly lower incidence rates among Korean adults with CKD, consisting of 15,443 CKD patients and 61,772 individuals without CKD from the nationwide healthcare database. K–M analysis, coupled with the log-rank test, consistently demonstrated a lower cumulative likelihood of newly occurring migraines over the 16-year monitoring period between the CKD group and the control group. To ensure data reliability, we utilized overlap-weighted models to meticulously balance baseline characteristics and took steps to address various potential confounding factors, including age, sex, income, and residence, as well as various lifestyle and comorbidity variables, by conducting multivariable conditional logistic regression to minimize the influence of confounding factors, including traditional vascular risk factors, as they can influence the occurrence of both CKD and migraine. We utilized the CCI score to evaluate the overall disease burden, which includes various comorbid conditions. Through this analysis, our study revealed a modest reduction of approximately 11% in the likelihood of migraine occurrence among CKD patients, especially among those with specific demographic characteristics such as older adults (aged 70 years or older), females, and those without hypertension. Considering the intricate challenges associated with managing migraines in individuals with renal disease [19], these findings may underscore the need for careful follow-up and preventive management for individuals lacking the identified protective factors, including male CKD patients under the age of 70 with hypertension. The observed modest reduction in migraine incidence among CKD patients may hold clinical significance, as it may offer insights into potential avenues for migraine management in this population. Understanding the factors contributing to this reduction could inform targeted interventions for migraine prevention and treatment among CKD patients.

Research into the relationship between CKD as a risk factor and incident migraines is currently lacking. Our study serves as the first population-based investigation dedicated to specifically examining the potential influence of CKD as a risk factor for the development of migraine. Most prior studies have focused primarily on investigating the reverse relationship—specifically, the impact of migraines as a risk factor for incident CKD [17,18]. In this context, a UK study utilizing UK Biobank observational data, which involved the analysis of genomic data from 48,975 migraine sufferers and 41,395 CKD patients, did not identify any causal effect of migraines on incident CKD [18]. However, a cross-trait meta-analysis revealed the presence of a genetic variant (rs1047891) that may have a shared influence on migraine, CKD, and renal function [18]. Transcriptome-wide association analysis revealed 28 gene expression correlations between migraine and renal function [18], suggesting an indirect and intricate association between CKD and migraine. Similarly, although some previous research suggested that individuals with CKD may experience migraines, the variations in migraine frequency did not reach statistical significance [30].

In our study, we found that certain CKD subgroups of older adults (aged 70 years or older) or females experienced a more pronounced reduction in migraine likelihood. There may be several potential interpretations for the notable decrease in the likelihood of migraine among CKD patients. The prevalence of migraine tends to decrease with age [31]. The incidence of migraine is lower among the elderly population (those aged above 60 years) than among younger individuals, with both incidence and prevalence declining as age increases [31]. The migraine predispositions depend weakly on age [31], although various risk factors have been proposed for migraines, including female sex, head trauma, lower socioeconomic status, caffeine or medication overuse, stress, sleep disturbances, obesity, pain syndromes, and proinflammatory or prothrombotic states [10]. Among the causes of migraine are factors such as changes in sleep patterns (e.g., insufficient sleep), alcohol consumption, and physical stress [32]. Although the prevalence of migraines in women is two to three times greater than that in men and estrogen has been linked to migraine susceptibility [33], CKD can induce sex hormonal dysregulation [34]. In women diagnosed with CKD, disruptions in the production of gonadotropin-releasing hormone can lead to an atypical sex hormone profile, ultimately resulting in reduced levels of estradiol [34]. This frequently leads to early menopause, menstrual irregularities, and infertility, affecting women with CKD [34].

Notably, our study revealed a reduced frequency of migraines among CKD patients, particularly those lacking traditional vascular risk factors such as hypertension. Hypertension is a prevalent comorbidity among CKD patients [3] and is associated with an elevated risk of migraines [10]. It is plausible that CKD patients in advanced stages may experience a greater incidence of migraines, possibly due to factors such as oxidative stress, uremic toxin accumulation, or fluctuations in blood pressure [35]. Since CKD and migraines share common contributing factors, such as menopause, smoking, alcohol consumption, and obesity [10,36], the absence of these shared risk factors may contribute to the decreased likelihood of migraines in CKD patients and the absence of this shared risk factor profile could contribute to the decreased likelihood of migraines in CKD patients. This may highlight the importance of considering individualized risk factor profiles in migraine management among CKD patients.

Recent data indicate that North America and high-income Asian countries, including Korea and Japan, demonstrate more successful hypertension control among CKD patients than other countries [37]. It is worth highlighting that a significant nationwide Korean cohort study focusing on outcomes in CKD patients in Korea reported the lowest prevalence of hypertension (27.3%) among the 17 global CKD cohorts that participated [37]. Given that the prevalence of CKD, migraine, and hypertension in CKD patients in Korea appears to be lower than those in other countries [8,37], the presence of hypertension among CKD patients in itself may have a more substantial impact on the likelihood of developing incident migraine. Conversely, the absence of hypertension in CKD patients could exert a protective influence on the occurrence of migraine. In general, disparities in health outcomes among populations with CKD may arise from variations in access to healthcare services and the quality of healthcare, in addition to factors such as environmental, nutritional, socioeconomic, and genetic influences [38]. In this context, the role of oxidative stress, uremic toxins, and fluctuations in blood pressure throughout the day in influencing migraine incidence among CKD patients may underscore the importance of addressing these factors in migraine management. Strategies aimed at mitigating oxidative stress and reducing uremic toxin accumulation could alleviate neurogenic inflammation associated with migraines during CKD. Similarly, interventions targeting blood pressure control may offer additional benefits in migraine prevention among CKD patients, particularly those with hypertension.

The reduced likelihood of migraines in CKD patients may also result from a multifaceted interplay involving shared factors and genetic susceptibility. Genomic studies have even revealed shared pathogenic mechanisms underlying both conditions; 11 single nucleotide polymorphisms (SNPs) have been identified that impact both migraine and renal function [18]. These SNPs exhibit pleiotropic characteristics, as seen in cross-trait meta-analyses between migraine and CKD, with diverse effects on the risks related to either condition [18]. These SNPs exert varying impacts on the risks associated with either migraine or CKD [18]. For instance, the SNP rs1047891, found in the *CPS1* gene, is linked to a reduced likelihood of migraine but an increased likelihood of CKD [18]. Despite its short half-life, *CPS1* appears to have anti-inflammatory properties independent of its enzymatic activity [39]. Recombinant *CPS1* has shown the ability to block liver injury and accelerate recovery in experimental mouse models, even when administered after injury [39,40]. This may suggest a nonenzymatic function of *CPS1* as an anti-inflammatory cytokine and provides a mechanism for its protective action, possibly reducing systemic inflammation and oxidative stress, which could protect against neurogenic inflammation associated with migraines during CKD.

The robustness and credibility of this study are grounded in the utilization of a comprehensive nationwide cohort database, which facilitated the achievement of balanced patient and control groups through the innovative technique of overlap-weighted propensity score matching. This approach aided in the reduction of selection bias and in the formation of study groups akin to those in randomized clinical trials [41], thereby enhancing the study’s credibility and robustness. While the earlier literature has highlighted a heightened prevalence of CKD in specific demographics, including men, elderly individuals, individuals with low income, and those residing in rural areas [17,18,42,43], our study successfully achieved a balanced distribution of demographic and health-related factors by precisely matching 15,443 individuals with corresponding control participants (*n* = 61,772) based on age, sex, income, and residential area. This enabled us to conduct a more precise examination of the relationship between CKD and migraines. Furthermore, the utilization of the KNHIS-HSC database in our study enabled the comprehensive retrieval of a complete medical history from every healthcare facility and clinic across the entire nation. This significantly improved the applicability and precision of our research findings. Third, another strength of our study was the careful consideration and adjustment for potential confounding variables, including socioeconomic factors, lifestyle-related risk factors, and comorbidities, enhancing the reliability and precision of our findings. Finally, our study was strengthened by a 16-year follow-up period, enabling a thorough and prolonged investigation into the CKD–migraine relationship.

Our findings must be considered with some constraints in mind due to their observational and retrospective design, which prevents the establishment of a causal relationship between CKD and migraines. Despite the common presence of vascular risk factors in CKD patients, particularly in advanced stages, our study was unfortunately limited by the available database, which lacks information on CKD stages, migraine severity, dialysis dependence, family history, genetics, medications, and dietary patterns. This limitation may have restricted our ability to determine the detailed relationship between early or later stages of CKD and incident migraines. Nonetheless, our findings consistently indicated a modest reduction in migraine incidence among overall patients with CKD throughout long-term follow-up, even after accounting for various confounding factors to the best of our ability. Based on our results, it appears that CKD may be less likely to increase the likelihood of migraine occurrence in the Korean adult population. Additionally, this study exclusively focused on Korean citizens aged 40 and older, utilizing diagnosis codes solely from Korean health insurance data, potentially leading to unaccounted-for confounding variables; its exclusive Korean patient cohort restricts the generalizability of the findings to other ethnic groups. We acknowledge that the correlation between CKD and incident migraines remains incompletely explained in our study.

## 5. Conclusions

Our study suggested a potential slight reduction in the likelihood of migraine development among Korean adults with CKD, particularly in certain subgroups. These findings emphasize the importance of vigilant follow-up and preventive management for migraine occurrence, especially among male CKD patients under the age of 70 with hypertension. Tailored approaches to migraine management in this population are warranted, taking into account individual risk factor profiles and potential underlying biological mechanisms. Further research is needed to validate these findings and elucidate the mechanisms driving this association.

## Figures and Tables

**Table 1:** Participant demographics.

Characteristics	Before Overlap-Weighting Adjustment			After Overlap-Weighting Adjustment		
	CKD	Control	Standardized Difference	CKD	Control	Standardized Difference
Age (y), *n* (%)			0.00			0.00
40–44	96 (0.62)	384 (0.62)		74 (0.65)	74 (0.65)	
45–49	355 (2.30)	1420 (2.30)		257 (2.25)	257 (2.25)	
50–54	896 (5.80)	3584 (5.80)		646 (5.65)	646 (5.65)	
55–59	1754 (11.36)	7016 (11.36)		1283 (11.23)	1283 (11.23)	
60–64	2146 (13.90)	8584 (13.90)		1566 (13.71)	1566 (13.71)	
65–69	2420 (15.67)	9680 (15.67)		1773 (15.52)	1773 (15.52)	
70–74	2687 (17.40)	10,748 (17.40)		1998 (17.49)	1998 (17.49)	
75–79	2581 (16.71)	10,324 (16.71)		1930 (16.89)	1930 (16.89)	
80–84	1681 (10.89)	6724 (10.89)		1261 (11.04)	1261 (11.04)	
≥85	827 (5.36)	3308 (5.36)		635 (5.56)	635 (5.56)	
Sex, *n* (%)			0.00			0.00
Male	10,415 (67.44)	41,660 (67.44)		7721 (67.59)	7721 (67.59)	
Female	5028 (32.56)	20,112 (32.56)		3702 (32.41)	3702 (32.41)	
Income, *n* (%)			0.00			0.00
1 (lowest)	2649 (17.15)	10,596 (17.15)		1945 (17.03)	1945 (17.03)	
2	1793 (11.61)	7172 (11.61)		1327 (11.62)	1327 (11.62)	
3	2207 (14.29)	8828 (14.29)		1629 (14.26)	1629 (14.26)	
4	3075 (19.91)	12,300 (19.91)		2269 (19.87)	2269 (19.87)	
5 (highest)	5719 (37.03)	22,876 (37.03)		4253 (37.23)	4253 (37.23)	
Region of residence, *n* (%)			0.00			0.00
Urban	6704 (43.41)	26,816 (43.41)		4958 (43.41)	4958 (43.41)	
Rural	8739 (56.59)	34,956 (56.59)		6465 (56.59)	6465 (56.59)	
Obesity ^†^, *n* (%)			0.16			0.00
Underweight	399 (2.58)	2006 (3.25)		312 (2.73)	312 (2.73)	
Normal	4735 (30.66)	22,085 (35.75)		3614 (31.64)	3614 (31.64)	
Overweight	4032 (26.11)	16,509 (26.73)		3009 (26.34)	3009 (26.34)	
Obese I	5576 (36.11)	19,449 (31.49)		4027 (35.26)	4027 (35.26)	
Obese II	701 (4.54)	1723 (2.79)		461 (4.03)	461 (4.03)	
Smoking status, *n* (%)			0.02			0.00
Nonsmoker	9726 (62.98)	39,481 (63.91)		7219 (63.19)	7219 (63.19)	
Past smoker	1674 (10.84)	6606 (10.69)		1248 (10.92)	1248 (10.92)	
Current smoker	4043 (26.18)	15,685 (25.39)		2957 (25.88)	2957 (25.88)	
Alcohol consumption, *n* (%)			0.07			0.00
<1 time a week	11,104 (71.90)	42,476 (68.76)		8119 (71.08)	8119 (71.08)	
≥1 time a week	4339 (28.10)	19,296 (31.24)		3303 (28.92)	3303 (28.92)	
SBP, mean (SD)	131.87 (18.47)	128.75 (16.37)	0.18	130.90 (15.46)	130.90 (7.31)	0.00
DBP, mean (SD)	78.78 (11.60)	78.16 (10.39)	0.06	78.57 (9.86)	78.57 (4.55)	0.00
Fasting blood glucose, mean (SD)	116.16 (50.06)	103.86 (29.10)	0.30	110.38 (33.22)	110.38 (17.83)	0.00
Total cholesterol, mean (SD)	190.31 (45.76)	193.28 (38.86)	0.07	190.68 (39.06)	190.68 (16.94)	0.00
CCI score, mean (SD)	2.19 (2.20)	1.11 (1.72)	0.54	1.83 (1.67)	1.83 (0.98)	0.00
Any migraine, *n* (%)	349 (2.26)	1901 (3.08)	0.05	261 (2.28)	347 (3.04)	0.05
Migraine with Aura	16 (0.10)	134 (0.22)	0.03	13 (0.11)	23 (0.20)	0.02
Migraine without Aura	333 (2.16)	1767 (2.86)	0.05	248 (2.17)	324 (2.84)	0.04

Abbreviations: CCI, Charlson Comorbidity Index; SBP, systolic blood pressure; DBP, diastolic blood pressure; CKD, chronic kidney disease; SD, standard deviation. ^†^ Obesity (body mass index, kg/m^2^) was categorized as underweight (<18.5), normal (18.5–23), overweight (23–25), obese I (25–30), or obese II (≥30).

**Table 2:** Crude and propensity score overlap-weighted HRs and 95% CIs of CKD for overall migraines, along with their subgroup analyses according to age, sex, income, and region of residence.

	N of Event/ N of Total (%)	Follow-Up Duration (PY)	IR per 1000 (PY)	IRD (95% CI)	HRs for Migraine			
					Crude	*p*	Overlap-Weighted Model ^†^	*p*
Total participants								
CKD	349/15,443 (2.26)	65,152	5.36	−0.62 (−1.27–0.02)	0.86 (0.77–0.97)	0.012 *	0.89 (0.81–0.97)	0.006 *
Control	1901/61,772 (3.08)	317,925	5.98		1		1	
Aged <70 years								
CKD	237/7667 (3.09)	42,360	5.59	0.07 (−0.70–0.86)	0.99 (0.86–1.14)	0.917	1.02 (0.91–1.13)	0.769
Control	1105/30,668 (3.60)	200,286	5.52		1		1	
Aged ≥70 years								
CKD	112/7776 (1.44)	22,792	4.91	−1.86 (−2.99 to −0.71)	0.68 (0.56–0.82)	<0.001 *	0.69 (0.60–0.80)	<0.001 *
Control	796/31,104 (2.56)	117,639	6.77		1		1	
Male								
CKD	193/10,415 (1.85)	42,922	4.50	−0.25 (−0.96–0.46)	0.91 (0.78–1.06)	0.234	0.93 (0.82–1.05)	0.222
Control	994/41,660 (2.39)	209,402	4.75		1		1	
Female								
CKD	156/5028 (3.10)	22,230	7.02	−1.34 (−2.64 to −0.04)	0.81 (0.69–0.96)	0.017 *	0.84 (0.73–0.95)	0.006 *
Control	907/20,112 (4.51)	108,523	8.36		1		1	
Low-income group								
CKD	153/6649 (2.30)	27,737	5.52	−0.83 (−1.85–0.19)	0.84 (0.70–0.99)	0.042 *	0.86 (0.76–0.98)	0.029 *
Control	874/26,596 (3.29)	137,732	6.35		1		1	
High-income group								
CKD	196/8794 (2.23)	37,415	5.24	−0.46 (−1.30–0.37)	0.89 (0.76–1.04)	0.130	0.90 (0.80–1.02)	0.087
Control	1027/35,176 (2.92)	180,193	5.70		1		1	
Urban resident								
CKD	152/6704 (2.27)	30,005	5.07	−0.32 (−1.23–0.58)	0.91 (0.76–1.08)	0.283	0.93 (0.81–1.06)	0.277
Control	777/26,816 (2.90)	144,150	5.39		1		1	
Rural resident								
CKD	197/8739 (2.25)	35,147	5.61	−0.86 (−1.77–0.05)	0.83 (0.72–0.97)	0.019 *	0.86 (0.76–0.96)	0.009 *
Control	1124/34,956 (3.22)	173,775	6.47		1		1	

Abbreviations: CKD, chronic kidney disease; IR, incidence rate; IRD, incidence rate difference; PY, person-year; HR, hazard ratio; CI, confidence interval. * Significance at *p* < 0.05. ^†^ Adjusted for age, sex, income, region of residence, obesity status, smoking status, alcohol consumption status, systolic blood pressure, diastolic blood pressure, fasting blood glucose, total cholesterol, and Charlson Comorbidity Index scores.

**Table 3:** Subgroup analyses of the crude and propensity score overlap-weighted hazard ratios and 95% confidence intervals of CKD for patients with migraine with aura.

	N of Event/ N of Total (%)	Follow-Up Duration (PY)	IR per 1000 (PY)	IRD (95% CI)	HRs for Migraine with Aura			
					Crude	*p*	Overlap-Weighted Model ^†^	*p*
Total participants								
CKD	16/15,443 (0.10)	66,982	0.24	−0.17 (−0.33 to −0.01)	0.55 (0.33–0.92)	0.024 *	0.63 (0.44–0.92)	0.016 *
Control	134/61,772 (0.22)	328,027	0.41		1		1	
Aged <70 years								
CKD	13/7667 (0.17)	43,686	0.30	−0.10 (−0.30–0.10)	0.71 (0.40–1.28)	0.261	0.82 (0.53–1.26)	0.363
Control	82/30,668 (0.27)	206,799	0.40		1		1	
Aged ≥70 years								
CKD	3/7776 (0.04)	23,296	0.13	−0.30 (−0.57 to −0.03)	0.27 (0.09–0.88)	0.029 *	0.30 (0.14–0.67)	0.003 *
Control	52/31,104 (0.17)	121,228	0.43		1		1	
Male								
CKD	10/10,415 (0.10)	43,836	0.23	−0.09 (−0.27–0.09)	0.66 (0.34–1.28)	0.219	0.73 (0.45–1.19)	0.208
Control	69/41,660 (0.17)	214,318	0.32		1		1	
Female								
CKD	6/5028 (0.12)	23,146	0.26	−0.31 (−0.63–0.01)	0.43 (0.19–1.00)	0.049 *	0.52 (0.29–0.93)	0.029 *
Control	65/20,112 (0.32)	113,709	0.57		1		1	
Low-income group								
CKD	10/6649 (0.15)	28,543	0.35	−0.14 (−0.42–0.13)	0.67 (0.34–1.29)	0.228	0.75 (0.46–1.22)	0.249
Control	70/26,596 (0.26)	142,342	0.49		1		1	
High-income group								
CKD	6/8794 (0.07)	38,439	0.16	−0.18 (−0.38–0.01)	0.43 (0.19–0.99)	0.047 *	0.50 (0.28–0.89)	0.018 *
Control	64/35,176 (0.18)	185,685	0.34		1		1	
Urban resident								
CKD	9/6704 (0.13)	30,786	0.29	−0.05 (−0.28–0.17)	0.80 (0.40–1.63)	0.544	0.92 (0.54–1.59)	0.777
Control	51/26,816 (0.19)	148,297	0.34		1		1	
Rural resident								
CKD	7/8739 (0.08)	36,196	0.19	−0.27 (−0.50 to −0.04)	0.39 (0.18–0.85)	0.018 *	0.45 (0.26–0.76)	0.003 *
Control	83/34,956 (0.24)	179,730	0.46		1		1	

Abbreviations: CKD, chronic kidney disease; IR, incidence rate; IRD, incidence rate difference; PY, person-year; HR, hazard ratio; CI, confidence interval. * Significance at *p* < 0.05. ^†^ Adjusted for age, sex, income, region of residence, obesity status, smoking status, alcohol consumption status, systolic blood pressure, diastolic blood pressure, fasting blood glucose, total cholesterol, and Charlson Comorbidity Index scores.

**Table 4:** Subgroup analyses of the crude and propensity score overlap-weighted hazard ratios and 95% confidence intervals of CKD for patients with migraine without aura.

	N of Event/ N of Total (%)	Follow-Up Duration (PY)	IR per 1000 (PY)	IRD (95% CI)	HRs for Migraine without Aura			
					Crude	*p*	Overlap-Weighted Model ^†^	*p*
Total participants								
CKD	333/15,443 (2.16)	65,241	5.10	−0.44 (−1.06–0.18)	0.89 (0.79–1.00)	0.05	0.90 (0.83–0.99)	0.029 *
Control	1767/61,772 (2.86)	318,765	5.54		1		1	
Aged <70 years								
CKD	224/7667 (2.92)	42,441	5.28	0.19 (−0.56–0.93)	1.02 (0.88–1.17)	0.829	1.03 (0.92–1.16)	0.581
Control	1023/30,668 (3.34)	200,857	5.09		1		1	
Aged ≥70 years								
CKD	109/7776 (1.40)	22,800	4.78	−1.53 (−2.63 to −0.43)	0.71 (0.58–0.86)	<0.001 *	0.72 (0.62–0.83)	<0.001 *
Control	744/31,104 (2.39)	117,908	6.31		1		1	
Male								
CKD	183/10,415 (1.76)	42,966	4.26	−0.15 (−0.84–0.54)	0.93 (0.79–1.09)	0.374	0.94 (0.83–1.07)	0.355
Control	925/41,660 (2.22)	209,834	4.41		1		1	
Female								
CKD	150/5028 (2.98)	22,275	6.73	−1.00 (−2.25–0.26)	0.84 (0.71–1.00)	0.055	0.86 (0.75–0.98)	0.024 *
Control	842/20,112 (4.19)	108,931	7.73		1		1	
Low-income group								
CKD	143/6649 (2.15)	27,803	5.14	−0.68 (−1.65–0.30)	0.85 (0.71–1.02)	0.079	0.87 (0.76–1.00)	0.051
Control	804/26,596 (3.02)	138,156	5.82		1		1	
High-income group								
CKD	190/8794 (2.16)	37,438	5.08	−0.25 (−1.07–0.55)	0.92 (0.79–1.08)	0.299	0.93 (0.82–1.05)	0.238
Control	963/35,176 (2.74)	180,609	5.33		1		1	
Urban resident								
CKD	143/6704 (2.13)	30,046	4.76	−0.27 (−1.14–0.61)	0.92 (0.77–1.10)	0.347	0.93 (0.81–1.07)	0.287
Control	726/26,816 (2.71)	144,470	5.03		1		1	
Rural resident								
CKD	190/8739 (2.17)	35,195	5.40	−0.57 (−1.45–0.30)	0.87 (0.75–1.02)	0.081	0.89 (0.79–1.00)	0.054
Control	1041/34,956 (2.98)	174,295	5.97		1		1	

Abbreviations: CKD, chronic kidney disease; IR, incidence rate; IRD, incidence rate difference; PY, person-year; HR, hazard ratio; CI, confidence interval. * Significance at *p* < 0.05. ^†^ Adjusted for age, sex, income, region of residence, obesity status, smoking status, alcohol consumption status, systolic blood pressure, diastolic blood pressure, fasting blood glucose, total cholesterol, and Charlson Comorbidity Index scores.

## Data Availability

All data are available from the National Health Insurance Sharing Service (NHISS) database (https://nhiss.nhis.or.kr; accessed on 1 February 2023). The NHISS allows access to all these data for any researcher who promises to follow the research ethics guidelines at a processing charge. If you wish to access the data of this article, you can download it from the website after promising to follow the research ethics.

## Supplementary Materials

The following supporting information can be downloaded at https://www.mdpi.com/article/10.3390/jpm14040356/s1. Supplementary Table S1: Subgroup analyses of crude and overlap propensity score weighted hazard ratios (95% confidence intervals) of CKD for overall migraines. Supplementary Table S2: Subgroup analyses of crude and overlap propensity score weighted hazard ratios (95% confidence intervals) of CKD for patients with migraine with aura. Supplementary Table S3: Subgroup analyses of crude and overlap propensity score weighted hazard ratios (95% confidence intervals) of CKD for patients with migraine without aura.
