# Supplementary material for: Associations between Chronic Kidney Disease and Migraine Incidence: Findings from a Korean Longitudinal Big Data Study
Source: J Pers Med. 2024 Mar 28;14(4):356. doi: 10.3390/jpm14040356 (PMC11051226; doi:10.3390/jpm14040356)
Supplement: Supplementary file 1 [file jpm-14-00356-s001.zip › jpm-2923618-supplementary.pdf]

Supplementary Table S1. Subgroup analyses of crude and overlap propensity score weighted hazard ratios (95% confidence interval) of CKD for overall migraines

	N of event / N of total (%)	Follow-up duration (PY)	IR per 1000 (PY)	IRD (95% CI)	Hazard ratios for migraine			
					Crude	P-value	Overlap weighted model †	P-value
Underweight								
CKD	8 / 399 (2.01)	1,220	6.56	0.40 (-4.32 to 5.12)	0.95 (0.45-2.01)	0.9	0.93 (0.55-1.58)	0.785
Control	54 / 2,006 (2.69)	8,766	6.16		1		1	
Normal weight								
CKD	105 / 4,735 (2.22)	19,220	5.46	-0.57 (-1.75 to 0.61)	0.87 (0.71-1.07)	0.193	0.91 (0.79-1.06)	0.23
Control	678 / 22,085 (3.07)	112,451	6.03		1		1	
Overweight								
CKD	97 / 4,032 (2.41)	18,056	5.37	-0.86 (-2.11 to 0.39)	0.84 (0.68-1.04)	0.115	0.85 (0.72-1.00)	0.049*
Control	541 / 16,509 (3.28)	86,810	6.23		1		1	
Obese								
CKD	139 / 6,277 (2.21)	26,656	5.21	-0.50 (-1.50 to 0.50)	0.88 (0.73-1.05)	0.162	0.89 (0.76-1.03)	0.116
Control	628 / 21,172 (2.97)	109,898	5.71		1		1	
Non-smoker								
CKD	249 / 9,726 (2.56)	42,535	5.85	-0.81 (-1.65 to 0.04)	0.85 (0.75-0.98)	0.021*	0.88 (0.79-0.98)	0.015*
Control	1,380 / 39,481 (3.50)	207,278	6.66		1		1	
Past and current smoker								
CKD	100 / 5,717 (1.75)	22,617	4.42	-0.29 (-1.26 to 0.69)	0.89 (0.72-1.11)	0.308	0.89 (0.76-1.06)	0.187
Control	521 / 22,291 (2.34)	110,647	4.71		1		1	
Alcohol consumption <1 time a week								
CKD	274 / 11,104 (2.47)	47,755	5.74	-0.83 (-1.62 to -0.04)	0.84 (0.74-0.96)	0.009*	0.87 (0.78-0.96)	0.006*
Control	1,452 / 42,476 (3.42)	221,082	6.57		1		1	
Alcohol consumption ≥1 time a week								
CKD	75 / 4,339 (1.73)	17,397	4.31	-0.33 (-1.42 to 0.77)	0.90 (0.71-1.15)	0.411	0.94 (0.79-1.13)	0.537
Control	449 / 19,296 (2.33)	96,843	4.64		1		1	
SBP < 140 mmHg and DBP < 90 mmHg								
CKD	225 / 10,098 (2.23)	41,001	5.49	-0.71 (-1.53 to 0.11)	0.85 (0.74-0.98)	0.026*	0.86 (0.77-0.95)	0.004*

Control	1,393 / 44,849 (3.11)	224,695	6.20		1		1	
SBP ≥ 140 mmHg or DBP ≥ 90 mmHg								
CKD	124 / 5,345 (2.32)	24,151	5.13	-0.32 (-1.35 to 0.72)	0.91 (0.75-1.11)	0.344	0.94 (0.80-1.10)	0.444
Control	508 / 16,923 (3.00)	93,230	5.45		1		1	
Fasting blood glucose < 100 mg/dL								
CKD	191 / 7,168 (2.66)	32,666	5.85	-0.71 (-1.65 to 0.23)	0.86 (0.74-1.00)	0.056	0.85 (0.76-0.95)	0.005*
Control	1,240 / 34,115 (3.63)	189,096	6.56		1		1	
Fasting blood glucose ≥ 100 mg/dL								
CKD	158 / 8,275 (1.91)	32,486	4.86	-0.27 (-1.13 to 0.60)	0.92 (0.77-1.09)	0.344	0.94 (0.82-1.08)	0.391
Control	661 / 27,657 (2.39)	128,829	5.13		1		1	
Total cholesterol < 200 mg/dL								
CKD	202 / 9,557 (2.11)	37,479	5.39	-0.44 (-1.29 to 0.41)	0.89 (0.76-1.03)	0.12	0.92 (0.82-1.04)	0.189
Control	1,033 / 36,303 (2.85)	177,197	5.83		1		1	
Total cholesterol ≥ 200 mg/dL								
CKD	147 / 5,886 (2.50)	27,673	5.31	-0.86 (-1.86 to 0.14)	0.84 (0.70-1.00)	0.049*	0.86 (0.75-0.97)	0.019*
Control	868 / 25,469 (3.41)	140,728	6.17		1		1	
CCI scores = 0								
CKD	113 / 4,527 (2.50)	21,109	5.35	-0.14 (-1.19 to 0.92)	0.96 (0.79-1.16)	0.667	0.96 (0.85-1.09)	0.544
Control	976 / 33,694 (2.90)	177,929	5.49		1		1	
CCI scores = 1								
CKD	57 / 2,613 (2.18)	10,574	5.39	-1.23 (-2.89 to 0.44)	0.78 (0.59-1.03)	0.085	0.82 (0.67-1.00)	0.054
Control	382 / 11,149 (3.43)	57,730	6.62		1		1	
CCI scores ≥ 2								
CKD	179 / 8,303 (2.16)	33,469	5.35	-1.25 (-2.26 to -0.25)	0.78 (0.66-0.92)	0.004*	0.83 (0.71-0.97)	0.018*
Control	543 / 16,929 (3.21)	82,266	6.60		1		1	

Abbreviation: CKD, chronic kidney disease; IR, incidence rate; IRD, incidence rate difference; PY, person-year; HR, hazard ratio; CI, confidence interval
*Significance at $p<0.05$. †Adjusted for age, sex, income, region of residence, obesity, smoking, alcohol consumption, systolic blood pressure, diastolic blood pressure, fasting blood glucose, total cholesterol, and Charlson Comorbidity Index scores

Supplementary Table S2. Subgroup analyses of crude and overlap propensity score weighted hazard ratios (95% confidence interval) of CKD for migraine with aura

	N of event / N of total (%)	Follow-up duration (PY)	IR per 1000 (PY)	IRD (95% CI)	Hazard ratios for Migraine with Aura			
					Crude	P-value	Overlap weighted model †	P-value
Underweight								
CKD	0 / 399 (0.00)	1,256	0.00	-1.11 (-2.96 to 0.73)	N/A		N/A	
Control	10 / 2,006 (0.50)	8,985	1.11		1		1	
Normal weight								
CKD	3 / 4,735 (0.06)	19,764	0.15	-0.28 (-0.58 to 0.02)	0.34 (0.10-1.08)	0.067	0.38 (0.18-0.79)	0.009*
Control	50 / 22,085 (0.23)	115,979	0.43		1		1	
Overweight								
CKD	7 / 4,032 (0.17)	18,566	0.38	0.05 (-0.25 to 0.33)	1.07 (0.47-2.44)	0.871	1.19 (0.62-2.26)	0.606
Control	30 / 16,509 (0.18)	89,755	0.33		1		1	
Obese								
CKD	6 / 6,277 (0.10)	27,396	0.22	-0.17 (-0.42 to 0.08)	0.52 (0.22-1.23)	0.138	0.69 (0.36-1.33)	0.27
Control	44 / 21,172 (0.21)	113,308	0.39		1		1	
Non-smoker								
CKD	12 / 9,726 (0.12)	43,940	0.27	-0.18 (-0.39 to 0.03)	0.58 (0.32-1.05)	0.072	0.67 (0.43-1.03)	0.071
Control	97 / 39,481 (0.25)	214,754	0.45		1		1	
Past and current smoker								
CKD	4 / 5,717 (0.07)	23,042	0.17	-0.16 (-0.40 to 0.09)	0.48 (0.17-1.36)	0.167	0.55 (0.26-1.13)	0.104
Control	37 / 22,291 (0.17)	113,273	0.33		1		1	
Alcohol consumption <1 time a week								
CKD	14 / 11,104 (0.13)	49,206	0.28	-0.18 (-0.38 to 0.02)	0.58 (0.33-1.01)	0.054	0.70 (0.46-1.05)	0.087
Control	106 / 42,476 (0.25)	229,050	0.46		1		1	
Alcohol consumption ≥1 time a week								
CKD	2 / 4,339 (0.05)	17,776	0.11	-0.17 (-0.43 to 0.09)	0.38 (0.09-1.58)	0.182	0.39 (0.15-0.99)	0.048*
Control	28 / 19,296 (0.15)	98,977	0.28		1		1	
SBP < 140 mmHg and DBP < 90 mmHg								
CKD	13 / 10,098 (0.13)	42,075	0.31	-0.17 (-0.40 to 0.05)	0.60 (0.34-1.07)	0.082	0.66 (0.44-0.98)	0.041*
Control	112 / 44,849 (0.25)	231,883	0.48		1		1	
SBP ≥ 140 mmHg or DBP ≥ 90 mmHg								
CKD	3 / 5,345 (0.06)	24,907	0.12	-0.11 (-0.31 to 0.09)	0.49 (0.15-1.64)	0.248	0.60 (0.24-1.48)	0.266
Control	22 / 16,923 (0.13)	96,144	0.23		1		1	
Fasting blood glucose < 100 mg/dL								
CKD	12 / 7,168 (0.17)	33,751	0.36	-0.09 (-0.33 to 0.15)	0.75 (0.41-1.38)	0.359	0.80 (0.52-1.24)	0.317
Control	88 / 34,115 (0.26)	195,984	0.45		1		1	

Fasting blood glucose ≥ 100 mg/dL								
CKD	4 / 8,275 (0.05)	33,231	0.12	-0.23 (-0.44 to -0.02)	0.32 (0.12-0.90)	0.03*	0.37 (0.18-0.76)	0.007*
Control	46 / 27,657 (0.17)	132,043	0.35		1		1	
Total cholesterol < 200 mg/dL								
CKD	10 / 9,557 (0.10)	38,492	0.26	-0.13 (-0.34 to 0.08)	0.62 (0.32-1.20)	0.157	0.74 (0.45-1.22)	0.238
Control	71 / 36,303 (0.20)	182,644	0.39		1		1	
Total cholesterol ≥ 200 mg/dL								
CKD	6 / 5,886 (0.10)	28,490	0.21	-0.22 (-0.48 to 0.03)	0.47 (0.20-1.08)	0.074	0.53 (0.30-0.94)	0.03*
Control	63 / 25,469 (0.25)	145,383	0.43		1		1	
CCI scores = 0								
CKD	6 / 4,527 (0.13)	21,680	0.28	-0.08 (-0.34 to 0.18)	0.75 (0.33-1.74)	0.507	0.74 (0.44-1.24)	0.252
Control	65 / 33,694 (0.19)	183,030	0.36		1		1	
CCI scores = 1								
CKD	4 / 2,613 (0.15)	10,850	0.37	-0.15 (-0.60 to 0.31)	0.66 (0.23-1.87)	0.431	0.70 (0.33-1.50)	0.357
Control	31 / 11,149 (0.28)	59,834	0.52		1		1	
CCI scores ≥ 2								
CKD	6 / 8,303 (0.07)	34,452	0.17	-0.28 (-0.51 to -0.03)	0.37 (0.16-0.87)	0.023*	0.44 (0.21-0.92)	0.028*
Control	38 / 16,929 (0.22)	85,163	0.45		1		1	

Abbreviation: CKD, chronic kidney disease; IR, incidence rate; IRD, incidence rate difference; PY, person-year; HR, hazard ratio; CI, confidence interval

*Significance at $p<0.05$. †Adjusted for age, sex, income, region of residence, obesity, smoking, alcohol consumption, systolic blood pressure, diastolic blood pressure, fasting blood glucose, total cholesterol, and Charlson Comorbidity Index scores

Supplementary Table S3. Subgroup analyses of crude and overlap propensity score weighted hazard ratios (95% confidence interval) of CKD for migraine without aura

	N of event / N of total (%)	Follow-up duration (PY)	IR per 1000 (PY)	IRD (95% CI)	Hazard ratios for Migraine without Aura			
					Crude	P-value	Overlap weighted model †	P-value
Underweight								
CKD	8 / 399 (2.01)	1,220	6.56	1.58 (-2.72 to 5.88)	1.18 (0.56-2.51)	0.666	1.26 (0.72-2.21)	0.426
Control	44 / 2,006 (2.19)	8,840	4.98		1		1	
Normal weight								
CKD	102 / 4,735 (2.15)	19,233	5.30	-0.27 (-1.40 to 0.87)	0.92 (0.74-1.13)	0.416	0.96 (0.82-1.12)	0.579
Control	628 / 22,085 (2.84)	112,776	5.57		1		1	
Overweight								
CKD	90 / 4,032 (2.23)	18,083	4.98	-0.89 (-2.11 to 0.31)	0.83 (0.66-1.03)	0.097	0.83 (0.70-0.98)	0.03*
Control	511 / 16,509 (3.10)	86,991	5.87		1		1	
Obese								
CKD	133 / 6,277 (2.12)	26,705	4.98	-0.32 (-1.29 to 0.65)	0.90 (0.75-1.09)	0.298	0.90 (0.77-1.05)	0.183
Control	584 / 21,172 (2.76)	110,158	5.30		1		1	
Non-smoker								
CKD	237 / 9,726 (2.44)	42,605	5.56	-0.61 (-1.42 to 0.20)	0.88 (0.76-1.01)	0.06	0.89 (0.80-0.99)	0.04*
Control	1,283 / 39,481 (3.25)	207,896	6.17		1		1	
Past and current smoker								
CKD	96 / 5,717 (1.68)	22,636	4.24	-0.13 (-1.07 to 0.82)	0.93 (0.75-1.16)	0.505	0.92 (0.78-1.09)	0.353
Control	484 / 22,291 (2.17)	110,869	4.37		1		1	
Alcohol consumption <1 time a week								
CKD	260 / 11,104 (2.34)	47,836	5.44	-0.63 (-1.40 to 0.13)	0.86 (0.76-0.99)	0.031*	0.88 (0.79-0.98)	0.017*
Control	1,346 / 42,476 (3.17)	221,745	6.07		1		1	
Alcohol consumption ≥1 time a week								
CKD	73 / 4,339 (1.68)	17,405	4.19	-0.15 (-1.21 to 0.91)	0.94 (0.73-1.20)	0.621	0.99 (0.82-1.19)	0.881
Control	421 / 19,296 (2.18)	97,020	4.34		1		1	
SBP < 140 mmHg and DBP < 90 mmHg								
CKD	212 / 10,098 (2.10)	41,071	5.16	-0.52 (-1.31 to 0.27)	0.88 (0.76-1.01)	0.073	0.88 (0.79-0.98)	0.019*
Control	1,281 / 44,849 (2.86)	225,416	5.68		1		1	
SBP ≥ 140 mmHg or DBP ≥ 90 mmHg								
CKD	121 / 5,345 (2.26)	24,170	5.01	-0.20 (-1.22 to 0.82)	0.93 (0.76-1.13)	0.472	0.95 (0.81-1.13)	0.568
Control	486 / 16,923 (2.87)	93,349	5.21		1		1	
Fasting blood glucose < 100 mg/dL								
CKD	179 / 7,168 (2.50)	32,734	5.47	-0.60 (-1.51 to 0.30)	0.87 (0.74-1.02)	0.086	0.86 (0.77-0.96)	0.008*
Control	1,152 / 34,115 (3.38)	189,679	6.07		1		1	
Fasting blood glucose ≥ 100 mg/dL								

CKD	154 / 8,275 (1.86)	32,507	4.74	-0.02 (-0.87 to 0.81)	0.97 (0.81-1.15)	0.71	0.98 (0.85-1.14)	0.831
Control	615 / 27,657 (2.22)	129,086	4.76		1		1	
Total cholesterol < 200 mg/dL								
CKD	192 / 9,557 (2.01)	37,522	5.12	-0.30 (-1.11 to 0.52)	0.91 (0.78-1.06)	0.224	0.94 (0.83-1.06)	0.298
Control	962 / 36,303 (2.65)	177,630	5.42		1		1	
Total cholesterol ≥ 200 mg/dL								
CKD	141 / 5,886 (2.40)	27,719	5.09	-0.61 (-1.58 to 0.35)	0.87 (0.73-1.04)	0.125	0.88 (0.77-1.01)	0.063
Control	805 / 25,469 (3.16)	141,135	5.70		1		1	
CCI scores = 0								
CKD	107 / 4,527 (2.36)	21,156	5.06	-0.05 (-1.07 to 0.97)	0.97 (0.80-1.19)	0.785	0.98 (0.86-1.11)	0.732
Control	911 / 33,694 (2.70)	178,319	5.11		1		1	
CCI scores = 1								
CKD	53 / 2,613 (2.03)	10,586	5.01	-1.05 (-2.64 to 0.54)	0.80 (0.60-1.06)	0.121	0.83 (0.67-1.03)	0.083
Control	351 / 11,149 (3.15)	57,916	6.06		1		1	
CCI scores ≥ 2								
CKD	173 / 8,303 (2.08)	33,499	5.16	-0.96 (-1.93 to 0.02)	0.81 (0.68-0.97)	0.018*	0.86 (0.73-1.01)	0.06
Control	505 / 16,929 (2.98)	82,530	6.12		1		1	

Abbreviation: CKD, chronic kidney disease; IR, incidence rate; IRD, incidence rate difference; PY, person-year; HR, hazard ratio; CI, confidence interval

*Significance at $p<0.05$. †Adjusted for age, sex, income, region of residence, obesity, smoking, alcohol consumption, systolic blood pressure, diastolic blood pressure, fasting blood glucose, total cholesterol, and Charlson Comorbidity Index scores
